# The role of lower urinary tract symptoms in fall risk assessment tools in hospitals: a review

**DOI:** 10.12688/f1000research.21581.1

**Published:** 2020-04-03

**Authors:** Saskia Roggeman, Jeffrey P. Weiss, Erik Van Laecke, Johan Vande Walle, Karel Everaert, Wendy F. Bower

**Affiliations:** 1 NOPIA research group, Urology Department, Ghent Universtity Hospital, Ghent, Belgium; 2 Faculty of Medicine and Health Sciences, Department of Human Structure and Repair, Ghent University, Ghent, Belgium; 3 Department of Urology, SUNY Downstate College of Medicine, Brooklyn, NY, USA; 4 Urology Department, Ghent University Hospital, Ghent, Belgium; 5 5. Faculty of Medicine and Health Sciences, Department of internal Medicine and Pediatrics, Ghent University, Ghent, 9000, Belgium; 6 Department of Pediatric Nephrology, Ghent University Hospital, Ghent, Belgium; 7 Department of Medicine and Aged Care, Royal Melbourne Hospital, Melbourne, Australia; 8 Faculty of Medicine, Dentistry and Health Sciences, The University of Melbourne, Melbourne, Australia

**Keywords:** in-hospital falls, Lower urinary tract symptoms, nocturia

## Abstract

A large number of falls in hospitals occur on the way to the toilet. Accordingly, a literature search was conducted in Web of Science, PubMed, Embase, and the Cochrane Library to identify fall risk screening and assessment metrics published between 1980 and 2019 and to study the inclusion of lower urinary tract symptoms (LUTS) and their related parameters in these screening tools. In addition, the literature was searched to explore the relationship between toilet-related falls and LUTS. In total, 23 fall risk scales were selected, from which 11 were applicable for in-hospital patients. In nine of the 11 scales for in-hospital patients, a LUTS or LUTS-related parameter was included. In the 12 risk assessment tools for community-dwelling older people, there were no LUTS included. Frequency, urinary incontinence, and nocturia were mostly reported in the literature as a potential fall risk parameter. It is recommended to create greater awareness of nocturia and other LUTS among caregivers of hospitalized patients to prevent falls.

## Introduction

Each year, at least 50% of people over 65 years of age will experience a fall. A fall, as defined by the World Health Organization, is “an event which results in a person coming to rest inadvertently on the ground or floor or other lower level”. Individuals who fall are generally frailer than those who don’t ^1^ and have a significantly longer in-hospital stay ^2^. Following a fall, especially against the backdrop of frailty markers, independence and functional ability at hospital discharge are compromised. Older patients with a history of a fall have a high risk of falling again; more than 20% of these patients will be hospitalized for a recurrent fall within 6 months ^3^, while an even greater proportion of patients will fall again within 12 months ^4^.

The risk of falls is attributable to the use of both multiple and specific medications, frailty or pre-frailty, depression, or incontinence ^5^. Many of the multiple causes of falls co-exist in older people hospitalized or in care centers ^6^. People with a slow walking speed are at particular risk of falls, suggesting that self-toileting may be an important fall-related activity in incontinent patients ^7^. Falls can occur at any age, but the frequency of falls increases with age ^8^. As a result, most fall risk tools are developed for older people. Accordingly, older patients with lower urinary tract symptoms (LUTS) should be identified on admission to the hospital ward in order to assess and prevent their risk of toileting-related falls.

LUTS are divided into three groups: storage, voiding, and post-micturition symptoms. When linked to falls, the symptoms related to storage are most relevant: increased daytime urinary frequency (voiding more than eight times a day), nocturia (waking to pass urine during the main sleep period), urinary urgency (sudden compelling desire to pass urine, which is difficult to defer), and urinary incontinence (any involuntary leakage of urine) ^9, 10^. One of the main causes of nocturia is nocturnal polyuria, often resultant from a disruption of the diurnal variation in secretion of arginine vasopressin that regulates resorption of water from the renal tubules ^11^. Individual urine production at night can easily be assessed in hospitalized patients using a fluid/volume chart or smart uroflow or containment product technology. Currently, the estimation of overnight urine production is not a standard procedure for patients with nocturia.

LUTS share common causal pathways with frailty ^4^. In particular, there is a significant association between slow walking speed and high fall risk in older adults ^7^. Urinary urgency has recently been shown to reduce walking speed by inducing shorter step length, greater gait variability, and a more flexed posture ^12^. The consequences of urinary urgency for safe mobilization at night in frail people are compounded by co-existing impaired balance, muscle weakness, and co-morbid illness that increases the rate of urine production. In fact, as many as 46% of all falls at night in subacute hospitalized patients occur on the way to the toilet ^13– 15^. Not surprisingly, the risk of injurious falls is significantly greater in patients who wake to void compared to people who sleep through the night (odds ratio [OR] 2.2, 95% confidence interval [CI] 1.04–3.87 for any fracture; OR 1.4, 95% CI 1.03–1.8 for hip fracture) ^16^. A recent systematic review concluded that nocturia increases the relative risk of falls by 20% and fractures by 32% ^17^. Falls and fractures arising from night toileting contribute to increased hospitalization and medical care costs ^18^.

Therefore, strategies that reduce the fall risks related to LUTS would have clinical utility, especially for patients who need to toilet during the night. Staff may potentially improve patient safety by identifying signs and/or symptoms of lower urinary tract dysfunction. The aim of this study was twofold: firstly, to identify which LUTS and LUTS-related factors are captured in fall risk assessment tools and, subsequently, to evaluate evidence of incorporating LUTS as a risk factor to potentially reduce fall risk while hospitalized.

## Materials and methods

A literature search was conducted in Web of Science, PubMed, Embase, and the Cochrane Library to identify fall risk screening and assessment metrics published between 1980 and 2019. Only papers in the English, Dutch, German, and French languages were considered for inclusion. Keywords used in the search were “fall risk assessment tool”, “fall risk scale”, “toileting management”, “fall prevention”, “nocturia”, “night-time falls”, and “in-hospital falls”.

The selected papers and reference lists were scrutinized to identify further fall risk assessment tools. Multiple fall risk scales described in the literature were searched for the inclusion of LUTS: urinary urgency, frequency, elimination, incontinence, and nocturia. Two extra LUTS-related parameters, intravenous infusion and use of diuretic medications, were also investigated given their impact on urine production. Findings were tabulated. The literature search also investigated the relationship between toilet-related falls and LUTS.

## Results

The literature search uncovered 23 fall risk scales. Of these, 11 were used for in-hospital patients or both in-hospital and institutionalized or community-dwelling older people ^19– 29^. The other 12 were intended for use in community-dwelling older people. None of the fall risk assessment tools for community-dwelling older people included LUTS or LUTS-related parameters. Instead, the focus was on direct measures of everyday activities and/or mobility and balance, i.e. walking speed. Fall risk tools for the in-hospital patients were applied by healthcare professionals and utilized clinical signs such as age, medication, and cognitive function.

When the selected fall risk tools for hospitals were searched for the inclusion of LUTS or LUTS-related risk parameters, urgency (two studies), frequency (two studies), elimination (three studies), incontinence (four studies), nocturia (one study), intravenous infusion (two studies), and diuretic use (four studies) were identified as risk factors for falls. In total, six out of the 11 fall risk scales included at least one question about LUTS. In most cases, the type of LUTS was directly mentioned in the question (e.g. are you incontinent?), but sometimes it was formulated by a question about their toileting (e.g. do you ever wet or soil yourself on the way to the bathroom?). In addition, five out of the 11 scales included the LUTS-related parameters infusion and diuretics ( Table 1). Of all risk assessment tools for hospitals, the Johns Hopkins Fall Risk Assessment Tool included most LUTS or LUTS-related parameters: i.e. urinary urgency, elimination, incontinence, infusion, and diuretics. The Falls Risk Assessment Tool (FRAT) examined urinary urgency, incontinence, nocturia, and diuretics and was therefore the only fall risk tool that included nocturia. The Falls Risk Assessment Score (FRAS) and the performance-based fall risk assessment tool did not include LUTS or LUTS-related parameters. The Morse, STRATIFY, and Conley fall risk scale included intravenous infusion, frequency, and incontinence, respectively ( Table 1).

**Table 1.** Overview of the selected fall risk scales for hospitals. Every scale was evaluated for the presence of lower urinary tract symptoms (LUTS) or LUTS-related parameters. B, bowel; U, urinary; U/B, no distinction made between urinary or bowel; X, included.

Fall risk tool	Urgency	Frequency	Elimination	Incontinence	Nocturia	Infusion	Diuretics	Reference
Conley Scale				U/B				^19^
Downton Fall Risk Index							X	^20^
Falls Risk Assessment Tool (FRAT)	U/B			U/B	X		X	^21^
Falls Risk Assessment Score (FRAS)								^22^
Hendrich II Fall Risk Model (HII-FRM)			U/B					^23^
Johns Hopkins Fall Risk Assessment	U/B		U/B	U/B		X	X	^24^
Medication Fall Risk Score & Evaluation Tools							X	^25^
MORSE Fall Scale						X		^26^
Performance-based fall risk assessment tool								^27^
Schmid Fall Risk Assessment Tool		U	U/B	U/B				^28^
St. Thomas Risk Assessment Tool in Falling Elderly Inpatients (STATIFY)		U/B						^29^

In the literature, the LUTS that were mostly reported to potentially increase the risk of falls were frequency, urinary incontinence ^30, 31^, and nocturia ^32^. During a study that compared the Morse Fall Scale, the Hendrich II Fall Risk Model, and the STRATIFY model, all parameters of the tools were tested together in a sample of Japanese inpatients. The inter-rater reliability and nurses’ perceptions of night-time toileting turned out to be among the poorest parameters, attributable to the unclear definition of frequency (e.g. urinary or bowel, number of voids/defecations) ^33^.

A 2003 evaluation of all fall risk scales published between 1980 and 2000 reported that only two LUTS-related parameters were commonly represented: elimination (18 of the 32 scales) and incontinence (11 of the 32 scales) ^30^.

The relationship between toilet-related falls and LUTS was described in several studies:

a 3-year prospective study of fall events in a German geriatric hospital reported 35–57 falls/1,000 hospital days. The majority of falls occurred in the patient’s room (73.5%) and in the toilet/bathroom (20%). The duration of in-hospital stay was longer for patients who had fallen ^34^. When the relationship and prevalence of toilet-related falls and LUTS were investigated, between 20 and 50% of falls in geriatric hospitals were associated with toileting and most often when the person was moving from the bedroom to the toilet or while using a bedside commode ^6^. Urinary incontinence proved to be a significant risk factor for falls in older people. At home, the highest risk of falls in the bathroom ^35^ occurred in people between 41 and 60 years of age. In patients with Parkinson’s disease, falls were related to urinary urgency but not to frequency of toileting ^36^.

A study of 447 hospitalized adults reported that 78% of them suffered from nocturia, 23% experienced urinary urgency, 22% had urinary incontinence, 11% had trouble passing urine, and 10% complained of fecal incontinence. The authors noted a lack of documented information about the continence status in the patient medical records and suggested that this led to an inaccurate estimation of the fall risk on the way to the toilet ^37^. Another study reported care professionals to have specified that patient diarrhea, frequent toileting, incontinence, and equipment connected to the patient (drainage, colostomy, etc.) were common causes of patient falls ^38^. Finally, sleep quality and sleep-disturbing factors in hospitalized patients were investigated; poor sleep quality was reported by 46% of patients, with frequent toilet use being the main cause of sleep disturbance ^39^. The authors suggested some strategies to minimize toilet use, such as dietary improvement, avoidance of excessive drinking, and reduction of treatment at night, e.g. change practice of night-time intravenous fluid infusions.

## Discussion

On the basis of a positive correlation between the prevalence of fall incidents and age, most fall risk tools have been developed for older people and predominantly focus on community-dwelling individuals. This review identified that fewer current measures are targeted towards the in-hospital population. The fall risk assessment tools for hospital populations differ from those for use in community-dwelling older people in both the items included and the data collection methods. In fall risk assessment tools for hospitals, the threat is assessed by the medical staff (e.g. nurses) using “clinical signs”. In contrast, the community-dwelling fall risk assessment tools use direct measures of everyday activities: mobility and balance (e.g. timed up and go, walking speed). No fall risk assessment tools for community-dwelling older people included LUTS or LUTS-related parameters. The likely rationale is that falls are multi-factorial and no single measure will capture the breadth of possible causes. Given that the reliability of measures directly relates to the intended clinical context, the selection of the optimal fall risk tool should be carefully considered ^40^.

This study identified a paucity of LUTS/LUTS-related factors included in fall risk assessment tools. Incontinence and elimination were the symptoms most commonly included in fall risk assessment tools for hospitals, being collected in four and three of the 11 tools, respectively. This finding was little changed from the systematic review conducted 17 years ago by Meyers ^30^. However, for all fall risk assessment tools from 1980 until now, no discrimination was made between urinary or bowel elimination and incontinence. Furthermore, there was no discrepancy between whether these risk factors appeared during the day or night. As urinary incontinence is a significant risk factor for falls in older people, it is recommended to screen for bladder dysfunction on hospital admission. Four fall risk tools included the use of diuretics as a risk factor, but only one metric considered toileting during the night.

Despite the inclusion of LUTS/LUTS-related contributors to fall risk, these parameters lacked standardized terminology in relation to LUTS. Factors such as “elimination” were vague and could not be quantified, i.e. did not specify the number of voids/day or urinary volume produced during sleep. There is no consensus in the literature about the sensitivity and specificity of the different fall risk assessment tools, particularly in relation to patients who void multiple times per night. This is likely attributable to confounding factors, covariance of parameters, and ward prevention measures that encourage containment products and discourage toileting.

Overall, the relationship between LUTS and falls is significant ^4^ but underrepresented as a standard parameter in fall risk scales. Future work should propose and justify tighter definitions for currently ambiguous parameters, capture the exact circumstances of a fall in patient records, and test associations with covariates, such as age and frailty status. An obvious omission from most risk assessment evaluation forms or medical records is documentation of what prompted the patient to get out of bed during the night prior to their fall. Impaired cognition and selected neurological disorders can precipitate wakefulness, as can sleep walking and cues for thirst and micturition. In order to provide appropriate preventive measures in the future and better adapt risk assessment tools to reality, this information should be collected.

It is clear that nocturia is an important risk factor for falls ^14, 15, 32, 39^. During the daytime, more personnel are available to supervise mobility and to assist patients in reaching the toilet. During the night, when nursing staff is reduced, patients generally wait longer for toileting assistance. This becomes critical if urinary urgency and urge incontinence co-exist. Accordingly, strategies to modify nocturia may prevent some night-time falls. An approach that improves awareness of the health and safety risks associated with nocturia, followed by the identification of possible causes of nocturnal polyuria and factors that disrupt sleep, is suggested. A hypothesis in relation to nocturia is that intravenous infusion may increase the risk of falls in two possible ways: the fluid load may induce nocturnal polyuria and, secondly, the infusion stand may cause the patient to trip. One possible strategy to decrease the number of night-time falls in hospitals is to prevent infusion-induced nocturnal polyuria overnight. Similarly, staff attention to minimizing late-night oral fluid intake in patients with known nocturnal polyuria may reduce the need to toilet during sleep. Future work to develop and test a package of care strategies that modify the risk of excessive urine production, sleep disturbance, and reduced bladder storage overnight is justified. The safety and efficacy of targeted and/or temporary intervention for bladder dysfunction in older hospitalized patients should be explored in prospective trials.

The gaps in practice and assumptions related to applying knowledge of LUTS to individualized care have been highlighted by this review. At this point in time, there are no clear recommendations for selecting fall risk assessment tools for use in hospitalized patients ^40– 43^. Clearly, this is a fertile area for future study and would address the recommendations from the 6th International Consultation on Incontinence for further research to be performed on the mechanisms, prevalence, incidence, and remission rates of LUTS in acutely hospitalized older people ^44^.

## Conclusion

While some LUTS-related parameters are incorporated into existing in-hospital fall risk assessment tools, there remains the need for improved discrimination between urinary and bowel elimination/incontinence and symptoms of storage and emptying. Despite studies reporting high night-time fall rates on the way to the toilet, only one fall risk assessment tool included nocturia. Greater awareness of the safety implications of LUTS such as urinary urgency and nocturia would influence choices and priorities of caregivers and simultaneously support the safety and dignity of hospitalized patients.
